# Dentistry as a Profession

**Published:** 1919-09-06

**Authors:** 


### DENTISTRY AS A PROFESSION.

The dental profession is one that offers many attractions to young men and women seeking a vocation of real usefulness and profit. Although modern requirements very properly demand from the dentist real medical preparation for his career, and, on the other hand, mechanical training and manipulative skill, there is plenty of scope both for those whose tastes are almost entirely mechanical or scientific, and for those to whom either the healing art, or preventive medicine, is more attractive. The relation between general diseases and dental diseases is very close indeed; and conservative dentistry worthy of the name needs, or at any rate is much helped by, a very thorough knowledge of bacteriology. The prosthetic side of the profession—and this is, of course, the most remunerative branch of dentistry—gives scope for highly developed mechanical knowledge, applied science, inventive ability, and even, in some of its branches, for the artistic faculty. Nor need the dentist do all his own work. He who prefers the surgical and medical side of his work can be assisted by a colleague whose tastes are mechanical; and even a man who dislikes all surgical work can employ skilled and qualified colleagues for this side, while yet using his abilities and applying his scientific knowledge to his cases. Moreover, the dental profession is far from being overcrowded. The young man or woman choosing dentistry as a profession has almost the certainty of a good living in prospect. One drawback there is, which has done much to prevent many men from entering the profession.

In the present state of the law any man can practise dentistry without any qualification or preparation whatsoever, so long as he does not use the title "Dentist," or "Dental Practitioner." It is nevertheless possible for the dental profession to do much towards remedying the evil by education of the public, by demanding an amendment of the law, and by so conducting the practice of their profession as to raise it in the eyes of the public above the level of the tooth-manufacturing companies whose advice and surgery consist, for the most part, in making room in the victim's mouth for the wares they supply. About two and a-half years ago the Lord President of the Council

(*Continued on page* 563.)

